# Natural reservoir *Rousettus aegyptiacus* bat host model of orthonairovirus infection identifies potential zoonotic spillover mechanisms

**DOI:** 10.1038/s41598-022-24673-w

**Published:** 2022-12-03

**Authors:** Amy J. Schuh, Brian R. Amman, Jonathan C. Guito, James C. Graziano, Tara K. Sealy, Shannon G. M. Kirejczyk, Jonathan S. Towner

**Affiliations:** 1 Viral Special Pathogens Branch, Division of High-Consequence Pathogens and Pathology, United States Centers for Disease Control and Prevention, Atlanta, GA USA; 2 United States Public Health Service Commissioned Corps, Rockville, MD USA

**Keywords:** Virology, Viral host response, Viral reservoirs, Virus-host interactions

## Abstract

The human-pathogenic Kasokero virus (KASV; genus *Orthonairovirus*) has been isolated from the sera of Egyptian rousette bats (ERBs; *Rousettus aegyptiacus*) captured in Uganda and unengorged *Ornithodoros (Reticulinasus) faini* ticks collected from the rock crevices of ERB colonies in South Africa and Uganda. Although evidence suggests that KASV is maintained in an enzootic transmission cycle between *O. (R.) faini* ticks and ERBs with potential for incidental virus spillover to humans through the bite of an infected tick, the vertebrate reservoir status of ERBs for KASV has never been experimentally evaluated. Furthermore, the potential for bat-to-bat and bat-to-human transmission of KASV is unknown. Herein, we inoculate two groups of ERBs with KASV; one group of bats is serially sampled to assess viremia, oral, fecal, and urinary shedding and the second group of bats is serially euthanized to assess virus-tissue tropism. Throughout the study, none of the bats exhibit overt signs of clinical disease. Following the detection of high KASV loads of long duration in blood, oral, fecal, and urine specimens collected from ERBs in the serial sampling group, all bats seroconvert to KASV. ERBs from the serial euthanasia group exhibit high KASV loads indicative of virus replication in the skin at the inoculation site, spleen, and inguinal lymph node tissue, and histopathology and in situ hybridization reveal virus replication in the liver and self-limiting, KASV-induced lymphohistiocytic hepatitis. The results of this study suggest that ERBs are competent, natural vertebrate reservoir hosts for KASV that can sustain viremias of appropriate magnitude and duration to support virus maintenance through bat-tick-bat transmission cycles. Viral shedding data suggests that KASV might also be transmitted bat-to-bat and highlights the potential for KASV spillover to humans through contact with infectious oral secretions, feces, or urine.

## Introduction

Bats have been implicated as natural reservoir hosts for many high-consequence, zoonotic RNA viruses, including coronaviruses^1,2^, filoviruses^3–5^, lyssaviruses^6^, and paramyxoviruses^7–9^. The devastating health, economic, and social consequences resulting from the coronavirus 2019 (COVID-19) pandemic caused by severe acute respiratory syndrome coronavirus 2 (SARS-CoV-2)^10^, a virus with an ancestral bat origin^11^, underscores the need to preemptively characterize RNA virus infection and transmission dynamics in their natural reservoir bat hosts to assess virus spillover risk and develop appropriate risk-reduction strategies.

The genus *Orthonairovirus* (family *Nairoviridae*) comprises 48 viruses, including the highly human-pathogenic Crimean Congo hemorrhagic fever virus, that are maintained in enzootic cycles involving tick and vertebrate hosts^12^. Eight of the orthonairoviruses, including Estero Real^13^, Gossas^14^, Issyk-Kul^15–18^, Kasokero^19,20^, Keterah^14,21^, Leopards Hill^22^, Uzun Agach^23,24^, and Yogue^14,25^ viruses, have been isolated from bats and/or their argasid tick ectoparasites. A mouse model of Leopards Hill virus infection demonstrated an acute, lethal disease resembling Crimean Congo hemorrhagic fever in humans^22^, and Issy-Kul^15–17^ and Kasokero^19^ viruses are known human pathogens that cause febrile illness, often progressing to prolonged systemic disease. Despite their public health importance, the infection and transmission dynamics of orthonairoviruses in their bat hosts are poorly understood. Of particular interest, is whether orthonairovirus-infected bat hosts can shed virus and represent direct virus spillover risks to humans.

Egyptian rousette bats (ERB; *Rousettus aegyptiacus*), cave-roosting frugivores with a geographic range extending north into Turkey, west into Senegal, south into South Africa, and east into Pakistan^26^, are natural reservoir hosts for Marburg (family *Filoviridae*, genus *Marburgvirus*; geographic range of known virus circulation includes sub-Saharan Africa)^3–5,27–29^, Ravn (family *Filoviridae*, genus *Marburgvirus*; geographic range of known virus circulation includes sub-Saharan Africa)^4,5^, and Sosuga (family *Paramyxoviridae*, genus *Pararubulavirus*; geographic range of known virus circulation includes Uganda)^9,30^ viruses. Kasokero virus (KASV) was first described in 1977 after scientists at Uganda Virus Research Institute isolated infectious virus from 2/74 (2.7%) serum samples collected from ERBs captured at Kasokero Cave, Uganda and detected virus-specific antibodies in 50/74 (67.6%) of the bat serum samples^19^. During initial virus characterization studies at the institute, 3 laboratory staff and 1 support staff acquired KASV infection, and presented with manifestations ranging in severity from mild febrile illness to prolonged systemic disease characterized by fever, headache, myalgia, arthralgia, abdominal pain, nausea, diarrhea, chest pain, coughing, and hyperactive reflexes^19^. The 4 KASV-infected staff seroconverted, and KASV antibodies were detected in 10 additional staff. KASV was later isolated from both engorged and unengorged argasid ticks (*Ornithodoros (Reticulinasus) faini*) collected from rock crevices of ERB colonies at Lanner Gorge Cave, South Africa in 1994–1995 and Python Cave, Uganda in 2017^20^. Consistent with the behavior of other tick species in the family Argasidae^31^, *O. (R.) faini* ticks emerge from rock crevices to rapidly feed (≤ 30 min) on roosting ERBs before retreating into the crevices. As a result, *O. (R.) faini* ticks have been observed only occasionally on wild-caught ERBs. In line with their rapid feeding ability, a previous blood meal analysis of *O. (R.) faini* tick pools collected from rock crevices of Python Cave revealed that 28.2% of the pools included ticks that had recently fed on ERBs (authors’ unpublished data).

Although evidence suggests that KASV is maintained in an enzootic transmission cycle between *O. (R.) faini* ticks and ERBs with potential for incidental virus spillover to humans through the bite of an infected tick^19,20^, the vertebrate reservoir status of ERBs for KASV has not been experimentally evaluated. Herein, we determine the potential for KASV to be transmitted tick-to-bat, bat-to-tick, bat-to-bat, and bat-to-human by experimentally inoculating groups of ERBs with KASV and testing blood, oral, fecal, and urine samples collected through 18 days post inoculation (DPI) for the presence of KASV. We then evaluate health status and virus-tissue tropism by performing clinical chemistry panels and assessing 14 tissue types collected from KASV-inoculated ERBs serially euthanized at regular intervals through 20 DPI.

## Methods

### Animal and biosafety procedures

A total of 24 captive-bred juvenile ERBs (5–7 m of age) from the ERB breeding colony^27^ were used in this study. All animal procedures were approved by the Institutional Animal Care and Use Committee (IACUC) at the United States Centers for Disease Control and Prevention (CDC) and performed according to the Guide for the Care and Use of Laboratory Animals^32^. The CDC is an Association for Assessment and Accreditation of Laboratory Animal Care fully accredited research facility. Reporting for this study was carried out in compliance with the ARRIVE guidelines. Although the Biosafety in Microbiological and Biomedical Laboratories Manual^33^ recommends that KASV work be performed under biosafety level (BSL) 2 containment, all procedures conducted with KASV or KASV-inoculated bats were performed under BSL-4 containment. All bats were housed in a climate controlled BSL-4 animal area, with a 12 h day/12 h night cycle. Bats were provided daily with their body mass in fresh fruit supplemented with protein/vitamin powder (Lubee Bat Conservancy, Gainesville, FL) and received water ad libitum.

### Experimental design, monitoring, and specimen collection

The 24 ERBs were divided into 2 study arms: 9 bats were assigned to the serial sampling arm and 15 bats were assigned to the serial euthanasia arm. Bats in the serial sampling arm were acclimated to the BSL-4 laboratory for 5 days before beginning the study. To mimic a tick bite, all bats were inoculated by the intradermal route in the subcaudal abdominal region under isoflurane anesthesia at 0 DPI. 7 bats (4 males and 3 females) received 4.0 log_10_ tissue culture infectious dose 50 (TCID_50_) of the UGA-Tick-20170128 strain of KASV (Vero E6 + 2 passages; mycoplasma-free; sequence identical to GenBank Accession Numbers MT309090, MT309094, and MT309097 [Vero E6 + 1]) prepared in 0.1 mL of sterile phosphate buffered saline (PBS), and 2 negative control (NEG CO) bats (2 males) received 0.1 mL of sterile PBS. The KASV inoculum dose was selected based on the estimated volume of saliva secreted by feeding *Ornithodoros* spp. ticks (10 µL)^34^ and the KASV titer of a pool of 5 unengorged *O. (R.) faini* ticks (4.1 log_10_TCID_50_ equivalents (eq)/10 µL). The KASV-inoculated bats were housed in a single flight cage maintained within a bio-flow isolator with HEPA-filtered inlet and exhaust air supplies (Duo-Flow Mobile Units, Lab Products Inc., Seaford, DE), while the NEG CO bats were housed in a non-human primate-sized cage maintained within a second bio-flow isolator unit. Temperatures, blood, duplicate oral swabs, rectal swabs, and urine (opportunistically) were collected from all bats daily (beginning at 0 DPI for temperatures and blood, and 1 DPI for oral swabs, rectal swabs, and urine) to the end of the study, while weights were taken on a weekly basis. At 18 and 20 DPI, the bats were euthanized by an overdose of isoflurane followed by cardiac exsanguination.

Blood was taken from the cephalic vein using a sterile lancet (C&A Scientific, Manassas, VA), polyester-tipped applicators (Fisher Scientific, Grand Island, NY) were used to swab the oral mucosa, and rectal swabs were obtained opportunistically at the time rectal temperatures were taken by repurposing the plastic thermometer probe cover (MABIS Healthcare, Waukegan, IL). Opportunistic urine collection was attempted by allowing a single bat to hang in a sterile mouse cage fitted with an inverted wire top (Thoren Caging, Hazleton, PA) and later collecting the accumulated urine. Aliquots of whole blood were used to monitor for viremia (KASV RNA by quantitative reverse transcriptase-polymerase chain reaction [qRT-PCR]) and antibody responses (anti-KASV IgG by indirect enzyme-linked immunosorbent assay [ELISA]). Oral swabs, rectal swabs and urine were used to detect virus shedding (KASV RNA by qRT-PCR).

Bats in the serial euthanasia arm were acclimated to the BSL-4 laboratory for 10 days before beginning the study. All bats were inoculated by the intradermal route in the subcaudal abdominal region under isoflurane anesthesia at 0 DPI. 12 bats (8 males and 4 females) received 4 log_10_TCID_50_ of the UGA-Tick-20170128 strain of KASV prepared in 0.1 mL of sterile PBS and 3 NEG CO bats (1 males and 2 females) received 0.1 mL of sterile PBS. The KASV-inoculated bats were housed in primate-sized cages according to the DPI of euthanasia (3, 6, 9, and 12; each euthanasia group included 2 males and 1 female) that were maintained within a bio-flow isolator unit, while the NEG CO bats were housed in a non-human primate-sized cage maintained within a second bio-flow isolator unit. All bats were euthanized by an overdose of isoflurane followed by cardiac exsanguination. Temperatures, weights, and blood were taken from all bats at 0 DPI and at euthanasia. Aliquots of whole blood collected at 0 DPI and at euthanasia were used to assess viremia and antibody responses. Serum collected at euthanasia was used to perform clinical chemistries and attempt virus isolation (if corresponding whole blood specimen was positive for KASV RNA). Tissues (skin at the inoculation site, skin distal to the inoculation site, axillary lymph node, salivary gland, gonads, inguinal lymph node, small intestine, liver, spleen, heart, lung, kidney, colon/rectum, and brain) collected at necropsy from all bats in the serial euthanasia arm and randomly-selected bats euthanized at 18 (n = 3 bats) and 20 DPI (n = 3 bats) from the serial sampling arm were tested for KASV RNA by qRT-PCR to determine virus-tissue tropism. Following necropsy, bat carcasses and remaining tissues were immersed in 10% neutral buffered formalin for a minimum of 7 days in BSL-4 containment, with a complete formalin replacement occurring at least 3 days prior to processing for histopathology.

### RNA extraction and qRT-PCR

RNA was extracted on the MagMAX Express-96 Deep Well Magnetic Particle Processor (Thermo Fisher Scientific, Waltham, MA) from blood, oral, rectal, and urine specimens using the MagMAX Pathogen RNA/DNA Kit (Thermo Fisher Scientific) and from homogenized tissues using the MagMAX Total RNA Isolation Kit (Thermo Fisher Scientific).

Reverse-transcribed KASV and ERB beta-2-microglobulin (B2M) RNA were detected on the ABI 7500 Real-Time PCR System (Thermo Fisher Scientific) using the SuperScript III Platinum One-Step qRT-PCR Kit (Thermo Fisher Scientific), with amplification primers and reporter probes targeting the nucleoprotein gene (forward primer: GGACATTGACTCTCAAACATC, reverse primer: GTCCAGGCACACTCATAAAT, probe: *FAM*-AGCAGTCAT-ZEN-CGCAGCCACCAGAAA-*IABkFQ*) and the ERB B2M gene (forward primer: CAGCAAGGACTGGTCTTTCTAT, reverse primer: CCTCCATGATGCTGGTTAGTT, probe: *FAM*-TTC ACA CGG/ZEN/CAG CTG TAC TCA TCC-*IABkFQ*), respectively. To account for variation in weight among the tissue specimens, KASV qRT-PCR cycle threshold (C_T_) values were normalized using ERB B2M qRT-PCR C_T_ values. Relative KASV TCID_50_eq/mL (blood, oral, rectal, and urine specimens) or g (tissue specimens) were interpolated from standard curves generated from serial dilutions of the titrated KASV UGA-Tick-20170128 strain spiked into appropriate biological specimens.

### Quantification of inequalities in viremia and viral shedding

As described previously^28^, Lorenz curves of cumulative percentage of the KASV-inoculated bat population versus cumulative percentage of KASV viremia, oral shedding, and fecal shedding ranked in descending order were constructed and Gini indices were calculated to quantify inequalities in viremia and/or viral shedding.

### Serology

KASV antigen lysate for the anti-KASV IgG indirect ELISA was prepared by inoculating monolayers of Vero E6 cells in 10, 850 cm^2^ roller bottles with 10 mL of diluted KASV UGA-Tick-20170128 strain at a multiplicity of infection of 0.03 and then incubating the bottles for 1 h at 37 °C. Following the addition of 150 mL maintenance media (Leibovitz's L-15 Medium, 2% heat-inactivated fetal bovine serum, 100 units/mL penicillin, 100 μg/mL streptomycin, 50 μg/mL gentamicin, and 2.50 μg/mL amphotericin B), the roller bottles were incubated at 37 °C. At 6 DPI, ≥ 90% of cells exhibited evidence of KASV infection by IFA.

At 7 DPI, glass beads were added to each roller bottle, the bottles were gently swirled to release virus-infected cells from tissue culture monolayers, and the cellular media was centrifuged at 8000 rpm for 10 min at 4 °C. Following centrifugation, the supernatant was discarded, cell pellets were resuspended in borate saline, and centrifuged at 12,000 rpm for 10 min at 4 °C. The cell pellets were resuspended in 28 mL of a 1% Triton X-100 borate saline solution, gamma-cell irradiated with a 5 megarad dose, and then sonicated for 15 min using a setting of 2 s on/2 s off. The sonicated KASV lysate was centrifuged at 12,000 rpm for 10 min at 4 °C and the supernatant was transferred to a fresh conical tube. Uninfected Vero E6 cell control lysate was prepared using the same procedure.

Wells of 96-well ELISA plates were coated (100 µL) with the dilution of KASV lysate (diluent: PBS containing 1% thimerosal) that was found to result in optimal reactivity with blood pooled from KASV-inoculated bats euthanized at 20 DPI (1:2000) and corresponding wells were coated with an equivalent dilution of uninfected control lysate (1:2000). After incubation overnight at 4 °C, the plates were washed with PBS containing 0.1% Tween-20 (PBS-T) and 100 µL of serum diluent (PBS containing 5% skim milk and 0.1% tween-20) was added to each well of the plate. After 10 min, 33 µL of a 21:521 dilution of gamma-irradiated bat serum pre-diluted in masterplate diluent (PBS containing 5% skim milk powder, 0.5% tween-20 and 1% thimerosal) was added to the first well of the plate and fourfold serial dilutions, ranging from 1:100 to 1:6400, were performed. Following a 1 h incubation at 37 °C, the plates were washed with PBS-T and 100 µL of a 1:20,000 dilution of goat anti-bat IgG conjugated to horseradish peroxidase (Bethyl Laboratories, Montgomery, TX; Cat#: A140-118P, Lot#: A140-118P-13) in serum diluent was added to the plates. The manufacturer product specification sheet indicates that this antibody reacts specifically with bat IgG and with light chains common to other immunoglobulins. After incubation for 1 h at 37 °C, the plates were washed with PBS-T, 100 µL of the Two-Component ABTS Peroxidase System (KPL, Gaithersburg, MD) was added, and the plates were allowed to incubate for 30 min at 37 °C. The plates were then read on a microplate spectrophotometer set at 410 nm. To negate non-specific background reactivity, adjusted optical density (OD) values were calculated by subtracting the ODs at each fourfold dilution of wells coated with uninfected control antigen lysate from ODs at corresponding wells coated with KASV lysate. The adjusted sum OD value was determined by summing the adjusted OD values at each four-fold serial dilution.

The cut-off value for seropositivity (0.27) was determined by calculating the mean adjusted sum OD value plus 5 standard deviations (SDs) of 38 KASV-naïve bats from the ERB breeding colony. A bat with an adjusted sum OD value 0.27 was considered to have been infected with KASV and seroconverted with a confidence level > 99.99999%.

### Virus isolation and immunofluorescence assay

Virus isolation was attempted on the serum fraction of all KASV RNA positive whole blood specimens collected at euthanasia. Monolayers of 85% confluent Vero E6 cells (American Type Culture Collection, CRL-1586; mycoplasma-free) in 25 cm^2^ tissue culture flasks were inoculated with 100 µL sera plus 500 µL maintenance media (Dulbecco's Modified Eagle Medium containing 2%, heat-inactivated fetal bovine serum, 100 units/mL penicillin, 100 μg/mL streptomycin, and 2.50 μg/mL amphotericin B) and incubated for 1 h at 37 °C/5% CO_2_. Following the addition of 7 mL maintenance media, cultures were incubated at 37 °C /5% CO_2_. At 13 DPI, tissue culture monolayers were scraped to release virus-infected cells. Part of each cellular medium (100 µL) was transferred to 500 µL MagMax lysis buffer solution for RNA extraction and qRT-PCR, and a second part of each cellular medium (1.5 mL) was suspended in 8 mL borate saline. After pelleting the cellular suspensions by centrifugation, the borate saline was decanted, the cells were resuspended in 500 μL borate saline, and 12-well spot slides were spotted with 25 μL of the cellular resuspensions. The slides were fixed in acetone before receiving 2 megarads of γ-irradiation.

Six spots on each slide were incubated with 25 μL of a 1:100 dilution of KASV mouse immune ascitic fluid (World Reference Center for Emerging Viruses and Arboviruses, Galveston, TX), and the other 6 spots were incubated with normal mouse ascitic fluid for 30 min at 37 °C. After incubation, the spot slides were rinsed 2 times with PBS, incubated with 24 μL of a 1:40 dilution of goat anti-mouse fluorescein isothiocyanate (MP Biomedicals, Santa Ana, CA) for 30 min at 37 °C, rinsed with PBS, stained with Eriochrome Black T, rinsed with PBS, and then observed under a fluorescence microscope.

### Histopathology and in situ hybridization

Histopathology was performed on 18 KASV-inoculated bats euthanized in groups of 3 at 3, 6, 9, 12, 18, and 20 DPI, as well as 3 NEG CO bats. A full set of tissues from each formalin-fixed carcass was collected for histologic examination, including representative sections from the brain, eye, heart, lungs, trachea, thymus, esophagus, thyroid glands, liver (4 non-contiguous sections including gall bladder), stomach, small intestines (duodenum with pancreas, jejunum, and ileum), large intestines, spleen, kidney, adrenal glands, salivary gland, submandibular lymph node, tongue, axillary, inguinal, sublumbar and mesenteric lymph nodes, skin from the inoculation site, patagium, skin from the chest region and pectoral muscle. Tissue sections were placed into cassettes and processed routinely for histological analyses at the University of Georgia Histology Laboratory. Representative formalin-fixed paraffin-embedded (FFPE) tissues were sectioned at 4–5 μm, mounted on glass slides, and stained with hematoxylin and eosin. Slides were reviewed in a blinded manner without knowledge of time point or infection status.

In situ hybridization was performed on FFPE liver tissues from a subset of KASV-inoculated bats (the three 3-DPI bats, in which KASV viral loads were highest for that tissue, and the 3 NEG CO bats) using the RNAscope® 2.5 HD Reagent Kit—RED (Advanced Cell Diagnostics, Newark, CA, USA) according to the manufacturer’s instructions. A custom anti-sense probe targeting the complimentary RNA sequence of the nucleoprotein gene of KASV (GenBank Accession Number: MT309090) was designed. To confirm adequate RNA integrity of the FFPE samples, an ERB-specific, medium-expressing housekeeping gene (peptidylprolyl isomerase B; GenBank Accession Number: XM_016141088.1) probe was used as a positive control. A probe targeting the dapB gene (Advanced Cell Diagnostics, Newark, CA, USA) was used as a negative control.

### Clinical chemistries

Serum collected at euthanasia from 18 KASV-inoculated bats euthanized in groups of 3 at 3, 6, 9, 12, 18, and 20 DPI, as well as 3 NEG CO bats, were analyzed on Piccolo Xpress chemistry analyzers (Abaxis, Union City, CA) using the General Chemistry 13 Panel.

ERB-specific reference ranges were established for glucose (GLU), blood urea nitrogen (BUN), creatinine (CRE), calcium (CA), albumin (ALB), total protein (TP), alanine transaminase (ALT), aspartate transaminase (AST), alkaline phosphatase (ALP), and total bilirubin (TBIL) using serum collected at euthanasia from 13 NEG CO bats used here and in previous studies^27,35^. Analyte data were assessed for conformation to a normal Gaussian distribution using the Shapiro–Wilk test (two-tailed P > 0.05). For normally distributed analytes (BUN, CRE, CA, ALB, TP, ALT, and ALP), 2.5% and 97.5% reference intervals were estimated using the mean plus two SDs. For non-normally distributed analytes (GLU, AST, and TBIL), data were log_10_-transformed to achieve normality, 2.5% and 97.5% reference intervals were estimated using the mean plus two SDs, and the reference intervals were back-transformed to their original units. Reference ranges could not be calculated for gamma-glutamyl transferase (GGT) and amylase (AMY), as these analytes were not previously measured in serum collected from the 13 NEG CO bats.

### Data and statistical analyses

The number of bats used in this study was based on the reproductive capacity of the ERB breeding colony. An effort was made to sex-match bats according to group. Investigators were not blinded during the study. No bats or individual data points were excluded from any analysis. Prism 9.0.0 software (GraphPad, La Jolla, CA) was used to perform all data management tasks, figure constructions, statistical analyses, and measures of central tendency (i.e., mean, range, SD).

## Results

### No overt signs of clinical disease

**Figure 1:**
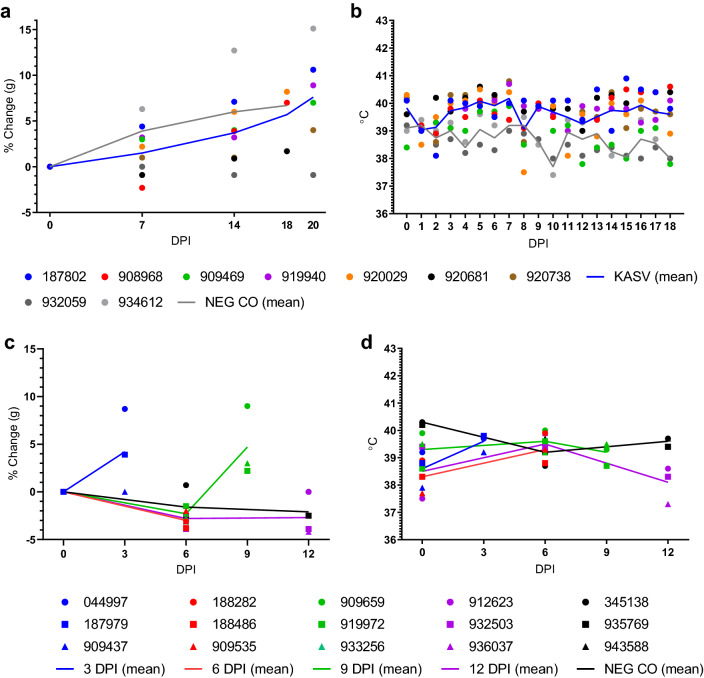
Weight and body temperature monitoring. (**a**) Percent weight change from baseline and (**b**) body temperatures of serially-sampled, Kasokero virus (KASV)-inoculated and negative control (NEG CO) bats, and (**c**) percent weight change from baseline and (**d**) temperatures of serially-euthanized, KASV-inoculated and NEG CO bats.

The KASV-inoculated bats in the serial sampling arm of the study gained less weight over time (Fig. 1a) and exhibited higher body temperatures throughout the study (Fig. 1b) compared to the NEG CO bats; however, the small bat group sizes precluded statistical comparisons and both parameters fell within the ranges previously observed for this bat species^27,28,30,35^. Like the serial sampling arm of the study, weights (Fig. 1c) and temperatures (Fig. 1d) of the bats in the serial euthanasia arm of the study were within the ranges previously observed for this bat species^27,28,30,35^. Neither the serial sampling nor serial euthanasia bats exhibited overt signs of clinical disease at any point during the study.

### KASV-inoculated bats mounted viremias and shed KASV through multiple routes

**Figure 2:**
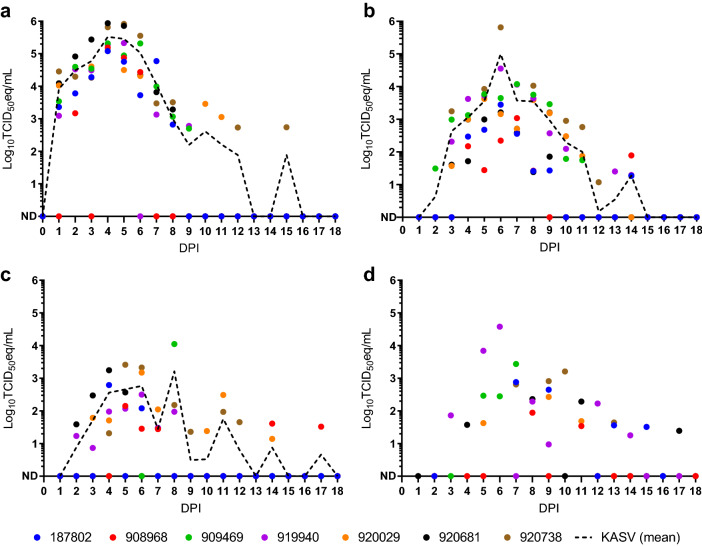
Kasokero virus (KASV) loads in specimens obtained from serially-sampled, KASV-inoculated bats. KASV loads (qRT-PCR-derived log_10_TCID_50_eq/mL) in (**a**) blood, (**b**) oral swabs, (**c**) rectal swabs, and (**d**) urine obtained from 0 to 18 days post inoculation (DPI). The dotted lines in panels a–c represent overall mean viral loads. The overall mean viral load for urine is not depicted in panel d, as this sample type was collected opportunistically, thereby biasing any measure of central tendency. ND: Not detected.

**Figure 3:**
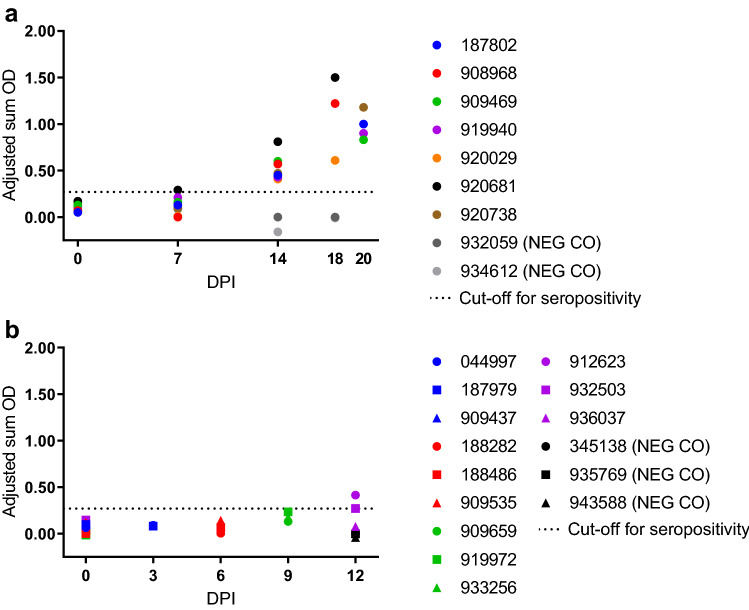
Anti-Kasokero virus (KASV) IgG responses. Anti-KASV IgG responses in (**a**) serially-sampled and (**b**) serially-euthanized, KASV-inoculated and negative control (NEG CO) bats according to days post inoculation (DPI).

Prior to inoculation, none of the bats in the serial sampling study arm (n = 9) had detectable viremias (Fig. 2a) or anti-KASV IgG (Fig. 3a), indicating no prior exposure to KASV. All specimens collected from NEG CO bats assigned to the serial sampling arm (n = 2) tested uniformly negative for KASV RNA (data not shown) and KASV IgG (Fig. 3a).

KASV viremia, and oral, fecal, and urinary shedding was detected in 100% (7/7) of the KASV-inoculated bats (Fig. 2). The interval of detectable viremia ranged from 1 to 15 DPI (mean duration = 7.7 days), with peak loads ranging from 5.1 to 5.9 log_10_TCID_50_eq/mL (mean peak load = 5.6 log_10_TCID_50_eq/mL) and occurring from 4–5 DPI (mean day of peak load = 4.3 DPI) (Fig. 2a). The interval of detectable oral shedding ranged from 2 to 14 DPI (mean duration = 8.6 days), with peak loads ranging from 3.0 to 5.8 log_10_TCID_50_eq/mL (mean peak load = 5.0 log_10_TCID_50_eq/mL) and occurring from 6–7 DPI (mean day of peak load = 6.1 DPI) (Fig. 2b). The interval of detectable fecal shedding ranged from 2 to 17 DPI (mean duration = 4.9 days), with peak loads ranging from 2.1 to 4.0 log_10_TCID_50_eq/mL (mean peak load = 3.2 log_10_TCID_50_eq/mL) and occurring from 4 to 8 DPI (mean day of peak load = 5.4 DPI) (Fig. 2c). Throughout the study, 126 opportunistic urine collection attempts resulted in 59 collections (collection proportion = 46.8%), which resulted in 27 KASV RNA detections (detection proportion = 45.8%). The interval of detectable urinary shedding ranged from 3 to 17 DPI, with peak loads ranging from 1.9 to 4.6 log_10_TCID_50_eq/mL and occurring from 6 to 10 DPI (Fig. 2d; mean urinary shedding duration and peak load were not calculated due to the opportunistic nature of the collections).

**Figure 4:**
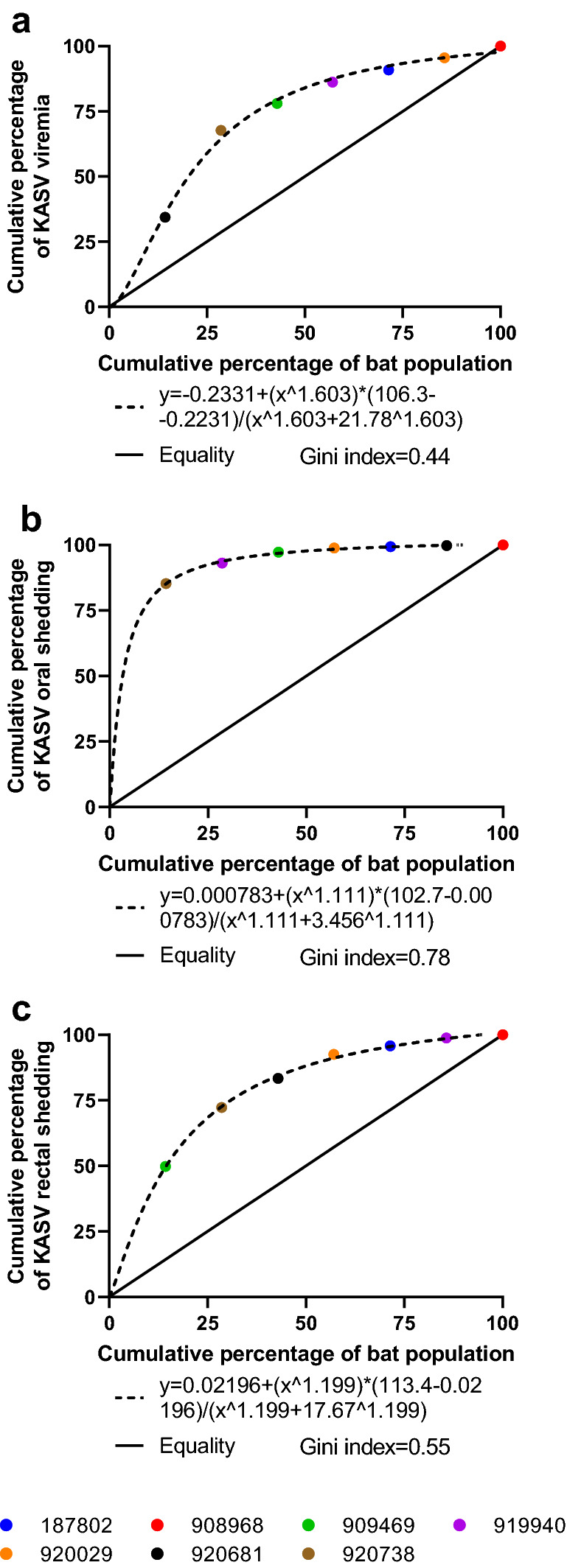
Cumulative Kasokero virus (KASV) shedding detected in specimens collected from serially-sampled, KASV-inoculated bats. Lorenz curves of cumulative percentage of the bat population versus cumulative percentage of KASV shedding detected in the (**a**) blood, (**b**) oral mucosa, and (**c**) rectum ranked in descending order (i.e., first symbol on the curve represents the bat which had the highest cumulative percentage of shedding).

Viremia and viral shedding are measures of infectiousness and were calculated for each KASV-inoculated bat by summing KASV RNA loads detected in the blood, oral and rectal swabs throughout the study^28,36^. Cumulative KASV RNA loads varied among the 7 KASV-inoculated bats, with sum log_10_TCID_50_eq/mL loads ranging from 5.4 to 6.3 (mean = 5.9, SD = 5.9) for blood specimens, 3.2–5.8 (mean = 5.1, SD = 5.4) for oral specimens, and 2.4–4.0 (mean = 4.3, SD = 3.6) for rectal specimens. The Lorenz curves for viremia (Fig. 4a), oral shedding (Fig. 4b), and fecal shedding (Fig. 4c) and their associated Gini indices show that a minority of the KASV-inoculated bat population was responsible for a disproportionate percentage of viral shedding. Following the Pareto Principle, the curve for oral shedding demonstrates that 20.0% of the KASV-inoculated bat was responsible for 89.9% of cumulative oral shedding. Two bats were classified as oral supershedders, as each of them orally shed KASV at loads greater than the 80th percentile (4.7 log_10_TCID_50_eq/mL) and together accounted for 93.1% of the total KASV oral shedding.

All KASV-inoculated bats (7/7) seroconverted to KASV by 14 DPI (Fig. 3a); anti-KASV IgG responses were highest at 18 or 20 DPI and coincided with the end of KASV shedding.

### KASV isolated from serum samples collected at euthanasia and high KASV loads detected in the skin at the inoculation site, spleen, and inguinal lymph node

Prior to inoculation, none of the bats in the serial euthanasia study arm (n = 15) had detectable viremias or anti-KASV IgG (Fig. 3b), indicating no prior exposure to KASV. All specimens collected from NEG CO bats assigned to the serial euthanasia study arm (n = 3) tested uniformly negative for KASV RNA (data not shown) and KASV IgG (Fig. 3b).

Blood collected from all bats euthanized at 3 and 6 DPI were KASV RNA positive and infectious KASV was isolated from serum collected from all bats euthanized at 3 DPI (Table 1). All 14 tissue types collected at necropsy were KASV RNA positive at some point during the study (Table 1). However, only tissues that exhibited viral loads greater than the corresponding blood specimen were considered KASV RNA positive, as the bats were not perfused before necropsy. Consistent with virus replication in the bat, KASV loads that exceeded viral loads in corresponding blood specimens and the inoculum dose (4.0 log_10_TCID_50_) were observed in the skin at the inoculation site (through 9 DPI), spleen (3 and 9 DPI), and inguinal lymph node (20 DPI).

**Table 1:**
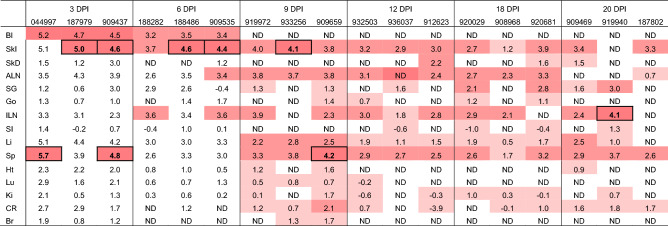
Kasokero virus (KASV) loads (qRT-PCR-derived log_10_TCID_50_eq/g) in tissues obtained from serially-euthanized, KASV-inoculated bats from 3–20 days post inoculation (DPI).

Boxes are shaded according to KASV qRT-PCR-derived log_10_TCID_50_eq/g loads: White: ND (not detected) or KASV load less than the load corresponding blood specimen, light pink: > 0.0– < 2.0 log_10_TCID_50_eq/g, medium pink: 2.0– 4.0 log_10_TCID_50_eq/g, and dark pink: > 4.0 log_10_TCID_50_eq/g. Outlined boxes with bolded values represent KASV loads that both exceed the inoculation dose (4.0 log_10_TCID_50_) and the corresponding KASV load in the blood, indicating the occurrence of virus replication.

*Bl* Blood, *SkI* skin at the inoculation site, *SkD* skin distal to the inoculation site, *ALN* axillary lymph node, *SG* salivary gland, *Go* gonads, *ILN* inguinal lymph node, *SI* small intestine, *Li* liver, *Sp* spleen, *Ht* heart, *Lu* lung, *Ki* kidney, *CR* colon and rectum, *Br* brain.

### KASV replicates in the liver and causes a self-limiting lymphohistiocytic hepatitis

**Figure 5:**
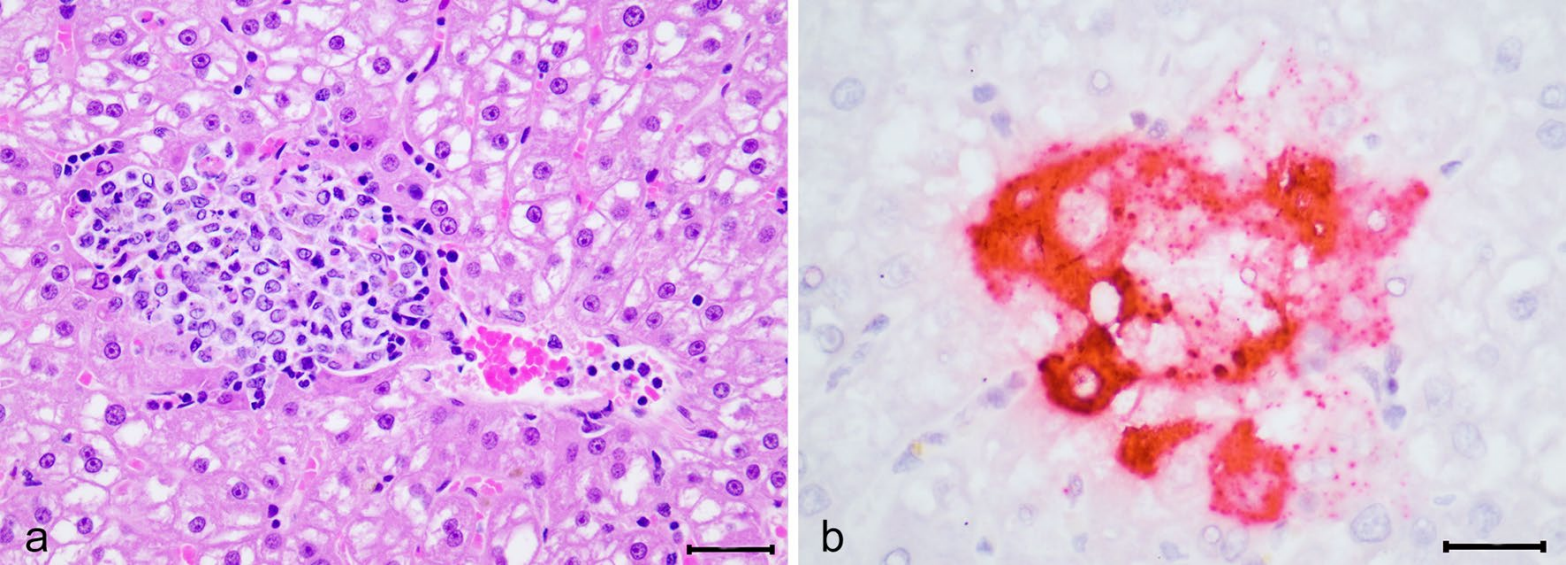
Histological lesions and localization of Kasokero virus (KASV) nucleoprotein RNA by in situ hybridization in a KASV-inoculated bat (044,997) euthanized at 3 days post inoculation. (**a**) A peri-venular aggregate of mononuclear inflammatory cells focally disrupts the hepatic parenchyma. Numerous degenerating hepatocytes are present along the periphery and mixed with the inflammatory cells. Hematoxylin and eosin stain, 40 ×, scale bar 20 µM. (**b**) Extensive positive hybridization signal for the KASV nucleoprotein gene is present in the cytoplasm of hepatocytes surrounding and within a focus of inflammation. Red chromogen with hematoxylin counterstain, 60 ×, scale bar 20 µM.

Gross examination at necropsy revealed a liver hemorrhage in one bat euthanized at 9 DPI (909,659). In the liver, KASV infection was associated with mild to moderate lymphohistiocytic hepatitis, which was histologically evident beginning at 3 DPI (Fig. 5). In situ hybridization demonstrated virus replication within the cytoplasm of hepatocytes, often associated with inflammatory cell infiltrates (Fig. 5, inset). Inflammatory cell foci were cleared from the liver by 20 DPI, consistent with a self-limiting acute viral hepatitis. The macroscopic liver hemorrhage observed at necropsy in bat 909,659 corresponded to a focal area of necrosis and hemorrhage. The NEG CO bats did not have any significant liver lesions, nor KASV RNA on histology and ISH, respectively (data not shown). Four KASV-inoculated bats had mild accumulations of macrophages and occasional neutrophils in the subcutaneous tissues adjacent to the KASV inoculation site (data not shown). There were no other significant lesions associated with KASV infection.

### Elevated liver enzymes indicative of acute viral hepatitis

**Figure 6:**
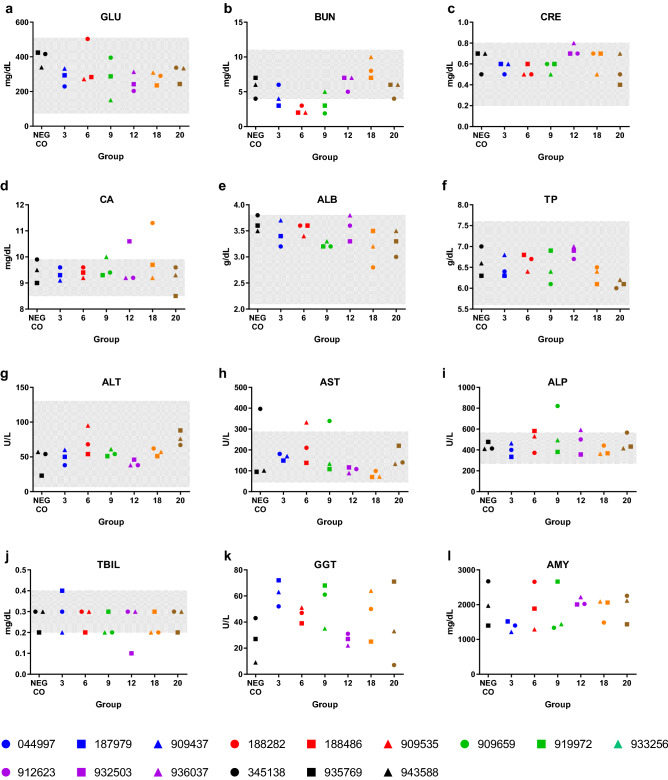
Clinical chemistry analytes measured in negative control (NEG CO) and Kasokero virus-inoculated bats euthanized at 3, 6, 9, 12, 18, or 20 days post inoculation. (**a**) glucose (GLU), (**b**) blood urea nitrogen (BUN), (**c**) creatinine (CRE), (**d**) calcium (CA), (**e**) albumin (ALB), (**f**) total protein (TP), (**g**) alanine transaminase (ALT), (**h**) aspartate transaminase (AST), (**i**) alkaline phosphatase (ALP), (**j**) total bilirubin (TBIL), (**k**) gamma-glutamyl transferase (GGT), and (**l**) amylase (AMY). Gray-shaded areas in (**a**–**j**) depict normal reference ranges.

Bat 909,659 exhibited elevated AST, ALP, and GGT (no reference range for GGT; relative to the NEG CO bats) liver enzymes (Fig. 6), which is indicative of acute viral hepatitis and consistent with the liver hemorrhage observed during necropsy. Other than 2 additional bats exhibiting elevated AST levels (345,138 [NEG CO] and 909,535 [6 DPI]) and 1 additional bat exhibiting elevated ALP levels (188,486 [6 DPI]), clinical chemistries were unremarkable.

## Discussion

The results of this experimental infection study combined with those of a previous ecological investigation of KASV circulation in ERBs at Kasokero Cave, Uganda^19^ suggest that ERBs are competent natural vertebrate reservoir hosts for KASV. Despite the detection of KASV in multiple tissues during this study and bats exhibiting a self-limiting KASV-induced lymphohistiocytic hepatitis, all bats displayed normal weights, body temperatures, and behavior, indicating the absence of significant clinical disease due to viral infection. Although we do not have a colony of *O. (R.) faini* ticks to experimentally evaluate bat-to-tick transmission of KASV, we demonstrated that ERBs experimentally inoculated with a tick isolate of KASV at a dose (4.0 log_10_TCID_50_) similar to that detected in a pool of unengorged *O. (R.) faini* ticks, mounted viremias that are likely of sufficient magnitude (mean peak load = 5.6 log_10_TCID_50_eq/mL) and duration (mean duration = 7.7 days) to infect feeding *O. (R.) faini* ticks. In line with a previous study that recovered infectious KASV from 2/74 (2.7%) serum specimens collected from ERBs captured at Kasokero Cave, Uganda^19^, we isolated infectious KASV from all serum specimens collected from bats euthanized at 3 DPI. Here, all KASV-experimentally infected ERBs in the serial sampling group seroconverted by 14 DPI and in the previous study at Kasokero Cave, virus-specific antibodies were detected in 50/74 (67.6%) of the ERB serum specimens^19^, indicating cumulative exposure of ERBs to KASV in nature. These data are consistent with the expectations of a natural vertebrate reservoir host of a virus.

In this study, KASV oral (mean peak load: 5.0 log_10_TCID_50_eq/mL; mean duration: 8.6 days), fecal (mean peak load: 3.2 log_10_TCID_50_eq/mL; mean duration: 4.9 days), and urinary (mean peak load: 4.3 log_10_TCID_50_eq/mL; mean duration: 3.9 days [opportunistic collection]) shedding was detected in all KASV-inoculated bats at some point during the study. Although KASV infection following mucosal virus inoculation of ERBs and/or experimental transmission of KASV from KASV-inoculated donor ERBs to naïve contact ERBs will be required to definitively demonstrate horizontal bat-to-bat transmission of KASV, our data indicate the potential for horizontal transmission of KASV between ERBs in nature through direct or indirect contact with infectious bodily fluids. KASV oral shedding dynamics were strikingly similar to those previously observed in MARV-subcutaneously inoculated ERBs (mean peak load: 4.7 log_10_TCID_50_eq/mL; mean duration: 4.6 days)^27,28^. Additionally, both KASV- and MARV-inoculated ERBs exhibited comparable heterogeneities in cumulative oral shedding, with 20% of the KASV and MARV study populations responsible for 89.8% and 95.4% of the viral oral shedding, respectively^28^. Based on the ability of the subcutaneous inoculation route to generate infection profiles that recapitulated MARV infection in ERBs in nature^5,27^ and the frequently observed altercations over roosting space in densely populated sub-Saharan African ERB colonies^5,26,27,37,38^, it was speculated that biting represents a primary route of bat-to-bat transmission of MARV^27^. As ERB bites presumably reach the intradermal and/or subcutaneous skin layers, horizontal transmission of KASV between ERBs through biting likely represents a viable transmission mechanism. Like KASV-experimentally infected ERBs, SOSV-experimentally infected ERBs inoculated through the subcutaneous route exhibited fecal (peak load: 3.9 log_10_TCID_50_eq/mL; detection range: 4–21 DPI) and urinary (peak load: 1.4 log_10_TCID_50_eq/mL; detection range: 8–21 DPI) shedding^30^. ERBs roosting in a densely populated colony at Python Cave, Uganda (~ 40,000 individuals) have been observed urinating and defecating just before and during flight, often when positioned directly above other bats, including virus-naïve juveniles, that frequently roost on vertical cave walls or the cave floor which is covered in guano^5^. Based on these observations and experimentally determined virus shedding routes, it was hypothesized that SOSV is horizontally transmitted between ERBs through direct or indirect contact with virus-contaminated urine and/or feces^30^. As KASV and SOSV exhibit comparable fecal and urinary shedding dynamics, it is likely that KASV can also be transmitted ERB-to-ERB through direct or indirect contact with urine and feces. Due to an unreported − 80 °C freezer failure, duplicate oral swab and urine specimens collected during this study for virus isolation attempts on KASV qRT-PCR positive specimens were lost. Nonetheless, persistently high KASV RNA loads in oral swab and urine specimens that occurred prior to the development of a robust virus-specific IgG response^28^ is consistent with virus replication in the host and indicates that virus isolation attempts would have likely resulted in the recovery of infectious virus.

The viremia, viral shedding, and virus-tissue tropism dynamics of KASV-experimentally infected ERBs suggest that KASV spillover into humans or wildlife could occur via multiple mechanisms. As the consumption of bushmeat is common in sub-Saharan Africa^29,39,40^, the detection of KASV in the blood and multiple tissues of KASV-experimentally ERBs indicates that preparing ERB meat for consumption or eating the undercooked meat may represent a risk factor for KASV infection. Likewise, consumption of KASV-infected ERBs by non-human primates or other animals could result in virus infection. Significant oral, fecal, and urinary shedding of KASV by experimentally infected ERBs suggest that consuming KASV-contaminated fruit or entering ERB roosts could result in KASV infection. ERBs forage for ripened fruit on a nightly basis, often test-biting and then dropping to the ground the fruit they decide not to consume^27^. After masticating fruit for consumption, ERBs squeeze the juice from the pulp using their palatal ridge and tongue, and then expectorate the pulp^37^. In light of these behaviors, plausible scenarios for KASV spillover to humans or wildlife may include the handling or consumption of ERB test-bitten fruit, fruit spats, or feces/urine-contaminated fruit. While the duration of KASV persistence on fruit is unknown, a recent study demonstrated that infectious MARV can persist on fruit for at least 6 h, a timespan encompassing the time from nightly ERB foraging for fruit to consumption of the fruit by humans or wildlife the next morning^41^. Although entering subterranean ERB roosts has not yet been associated with KASV infection, index cases of MARV outbreaks have reported entering ERB-occupied caves or their peripheries in the absence of acquiring any bat-inflicted bites or injuries^4,42–46^. Large amounts of feces and urine are released upon mass exit of ERBs from their roosts for nightly foraging or when colonies are disturbed by humans or wildlife. These lines of evidence indicate that ERB roosts are conducive environments for virus spillover via airborne or droplet transmission mechanisms.

In conclusion, the results of our study suggest that in addition to transmission by *O. (R.) faini* ticks, KASV has the potential be transmitted between ERBs and from ERBs to humans and wildlife through direct or indirect contact with infectious bodily fluids. Given that the known geographic range of KASV circulation and spillover risk extends from Uganda to South Africa, future investigations are needed to: 1) detect and isolate KASV from oral, rectal, and urine specimens from wild-caught ERBs and 2) determine if peaks of active KASV infection occur in young juveniles coincidental with the loss of maternal immunity similar to MARV infection^5^. Given that a mouse model of Leopards Hill virus infection generates a lethal disease resembling Crimean Congo hemorrhagic fever in humans^22^, and the extensive geographic range (Central Asia to Western Europe), outbreak history and disease profile of Issyk-Kul virus^15–18^, these two orthonairoviruses and the five other understudied bat-associated orthonairoviruses merit further investigation. Finally, the KASV-ERB system could serve as an experimental natural reservoir host model for studying the zoonotic potential of other bat-borne orthonairoviruses.

## Data Availability

All data generated or analyzed during this study are included in this published article.
